# An exosome-inspired docetaxel prodrug nanoplatform for potent STING activation and synergistic chemoimmunotherapy

**DOI:** 10.1016/j.ajps.2026.101139

**Published:** 2026-02-27

**Authors:** Xinying Wang, Xianlu Zhang, Zixuan Jiao, Shipeng Ning, Hanping Wang, Hui Liu, Lifen He, Haonan Li, Mingzhong Li, Kaiyuan Wang, You Pan, Zhonggui He

**Affiliations:** a Department of Pharmaceutics, Wuya College of Innovation, Shenyang Pharmaceutical University, Shenyang 110016, China; b Joint International Research Laboratory of Intelligent Drug Delivery Systems of Ministry of Education, Shenyang Pharmaceutical University, Shenyang 110016, China; c Department of Breast Surgery, The Second Affiliated Hospital of Guangxi Medical University, Nanning 530000, China; d Departments of Diagnostic Radiology, Surgery, Chemical and Biomolecular Engineering, and Biomedical Engineering, Yong Loo Lin School of Medicine and College of Design and Engineering, National University of Singapore, Singapore 119074, Singapore; e School of Pharmacy, De Montfort University, The Gateway, Leicester, LE1 9BH, UK; f Department of Urology, The First Hospital of China Medical University, 155 North Nanjing Street, Shenyang 110000, China; g Hainan Branch, Shanghai Children’s Medical Center, School of Medicine, Shanghai Jiao Tong University, Sanya, China

**Keywords:** Exosome-mimetic, STING activation, Docetaxel prodrug, Chemoimmunotherapy

## Abstract

The treatment of triple-negative breast cancer (TNBC) is significantly hampered by its immunosuppressive tumor microenvironment and limited T-cell infiltration. Activating the STING pathway presents a promising therapeutic avenue due to its potential to trigger a robust innate immune response and remodel the immunosuppressive landscape, thereby sensitizing tumors to immunotherapy. To harness this potential, we developed an exosome-mimetic nanoplatform (EMMDs). EMMDs is fabricated by co-loading a disulfide-linked docetaxel prodrug (DTX-SS-PA) and the STING agonist MSA-2 into polymeric micelles and further decorated with homotypic tumor cell-derived exosomal membrane (EM). This biomimetic design confers superior tumor-targeting capability. Upon reaching the tumor site, the prodrug is specifically activated to release cytotoxic docetaxel (DTX), while MSA-2 is concurrently released to potently activate the STING pathway. This dual action initiates a powerful antitumor immune response and reverses immunosuppression, leading to a synergistic chemo-immunotherapeutic outcome. In the murine TNBC model, EMMDs demonstrated remarkable antitumor efficacy, obviously provoking a robust STING-mediated type I interferon response and inhibiting tumor growth. This work presents a promising biomimetic strategy for remodeling the tumor immune microenvironment via efficient STING activation.

## Graphical abstract

DTX-SS-PA and STING agonist MSA-2 were co-loaded into polymeric micelles to form a nanocore, which is subsequently encapsulated with a 4T1 cancer cell-derived exosomal membrane to construct the biomimetic nanomedicine EMMDs. The resulting EMMDs effectively activate the STING pathway and synergize with chemotherapy to achieve potent chemoimmunotherapeutic effects against cancer.

**Figure:**
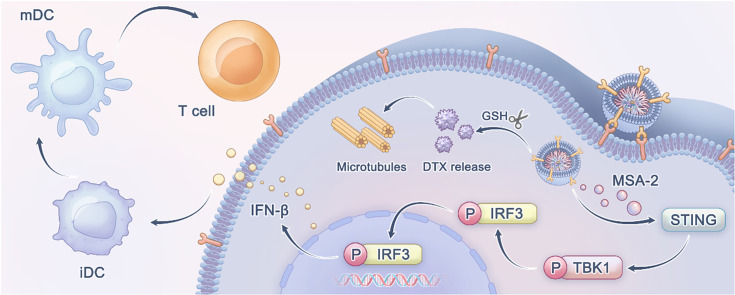

## 1 Introduction

Cancer remains a major global health challenge despite the availability of multiple therapeutic approaches [1,2]. Chemotherapy is still used as a first-line treatment for many cancers, yet its clinical efficacy is frequently compromised by systemic toxicity, multidrug resistance, and limited tumor selectivity [3]. To overcome these drawbacks, prodrug strategies have been developed to improve the physicochemical properties of anticancer agents, particularly their poor solubility, nonselective toxicity, and inadequate stability. Nanoplatform-based delivery systems have also attracted considerable attention in cancer therapy. Although substantial progress has been achieved, their clinical translation continues to face practical constraints. Issues such as formulation complexity, limited scalability, and batch-to-batch variability complicate reliable manufacturing and quality control. In addition, interactions between nanomaterials and the biological environment can influence *in vivo* behavior, leading to inconsistencies in therapeutic outcomes. These factors emphasize the need to balance functional performance with pharmaceutical feasibility in nanomedicine design. Nevertheless, challenges related to systemic clearance, biocompatibility, and suboptimal immune responses remain major obstacles to the clinical application of current nanoplatforms [4].

The stimulator of interferon genes (STING) is an intracellular sensor that plays an essential role in innate antitumor immunity. Upon activation, the STING pathway triggers the production of type I interferons and proinflammatory cytokines, thereby contributing to the remodeling of the immunosuppressive tumor microenvironment (TME). Previous studies have shown that activation of this pathway in tumor cells and dendritic cells (DCs) enhances antigen cross-presentation to CD8⁺ T cells, leading to the induction of tumor-specific T cell responses and strengthened antitumor immunity. Because of these immunostimulatory effects, STING agonists have attracted increasing interest as potential immunotherapeutic agents. They are capable of promoting DC maturation and amplifying antitumor T cell activity, while their incorporation into nanoplatforms further enables localized immune activation and reduces systemic toxicity [5,6]. MSA-2, a small-molecule STING agonist, stimulates type I interferon production and inflammatory cytokine release, thereby activating innate immunity and modulating the TME [[7], [8], [9]]. Although it shows certain therapeutic efficacy in animal models regardless of oral or intravenous administration, its limited bioavailability and insufficient cytoplasmic delivery efficiency still limit its therapeutic effect. Therefore, achieving precise and efficient delivery of STING agonists is a key prerequisite for fully exerting their antitumor potential.

A promising strategy is to cloak synthetic nanoparticles with naturally derived exosomal membrane (EM). This biomimetic modification improves biocompatibility, prolongs systemic circulation, and reduces immune recognition, while retaining intrinsic homing properties [[10], [11], [12]]. At the structural level, EM consists of a lipid bilayer enriched in cholesterol, sphingomyelin, and tetraspanins such as CD9 and CD81, which contribute to membrane stability and functional integrity [13]. Rather than acting solely as a passive coating, the EM also serves as a signaling interface. Its molecular constituents preserve biological activity and enable surface proteins to mediate specific cell recognition, thereby facilitating intercellular communication and regulating recipient cell functions [14]. In addition, membrane-associated self-markers transmit a “don’t eat me” signal that limits phagocytic clearance and extends circulation time [[15], [16], [17]]. Owing to these properties, EM provides a naturally derived delivery platform with inherent targeting potential. In particular, membranes isolated from murine triple-negative breast cancer (TNBC) cells retain parental molecular signatures that confer homologous tumor-targeting capability [[18], [19], [20]], promoting preferential recognition and uptake by the original cancer cells [21]. When coupled with high drug-loading capacity, this approach yields nanoparticles that evade immune clearance, achieve selective tumor accumulation, and undergo efficient cellular internalization, thereby enhancing therapeutic efficacy while reducing systemic toxicity.

**Scheme 1:**
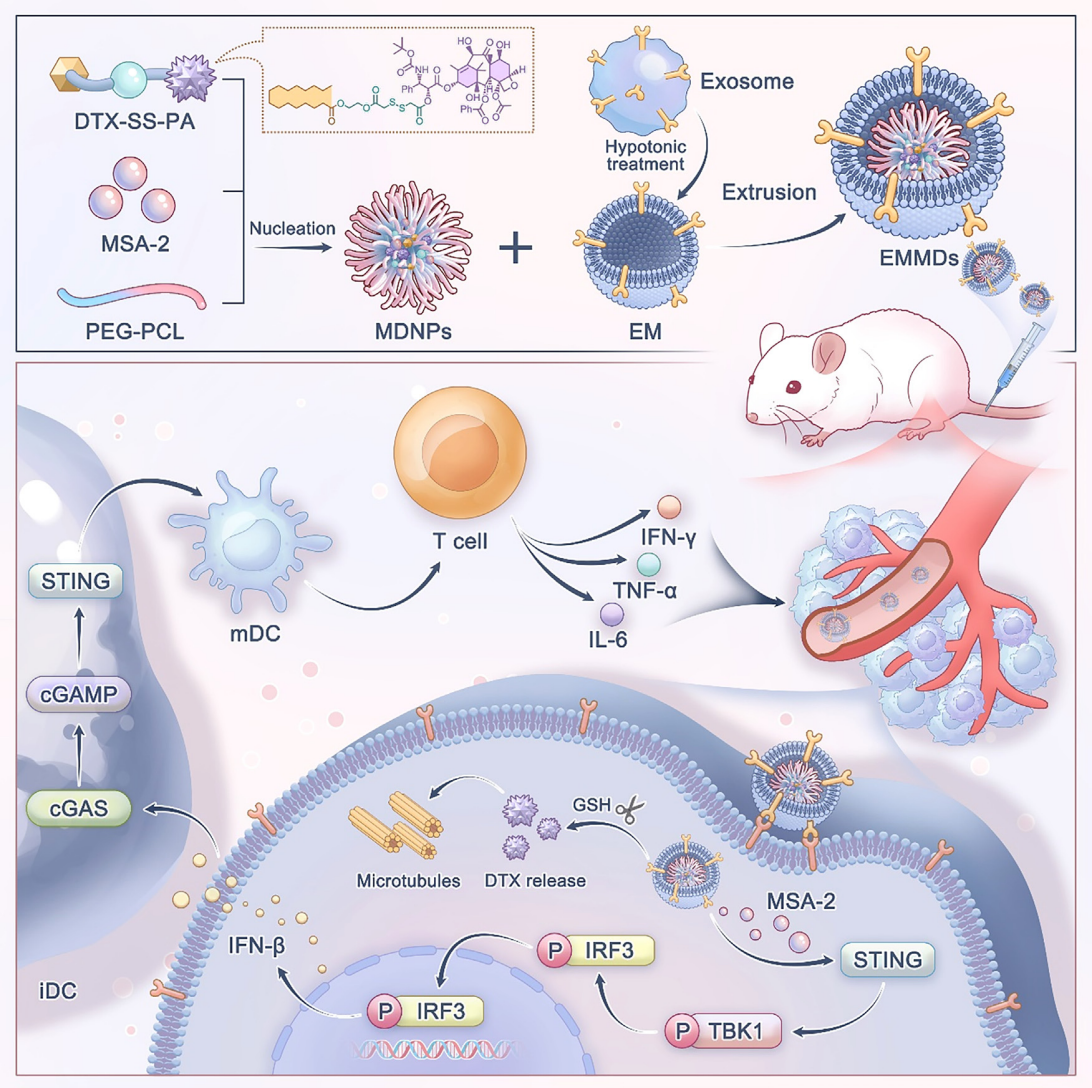
Schematic diagram. DTX-SS-PA and STING agonist MSA-2 were co-loaded into polymeric micelles to form a nanocore, which is subsequently encapsulated with a 4T1 cancer cell-derived EM to construct the biomimetic nanomedicine EMMDs. The resulting EMMDs effectively activate the STING pathway and synergize with chemotherapy to achieve potent chemoimmunotherapeutic effects against cancer.

To test this hypothesis, MSA-2 and DTX-SS-PA were co-encapsulated into polymeric micelles and subsequently coated with EM derived from 4T1 tumor cells, yielding EMMDs. Palmitic acid (PA), a naturally occurring C16 saturated fatty acid with favorable biocompatibility and a well-defined metabolic pathway, has been widely applied in prodrug construction and lipid-based drug delivery systems. From a formulation standpoint, PA provides sufficient hydrophobic interaction to drive the stable self-assembly of docetaxel (DTX) prodrugs, while avoiding excessive crystallization or overly delayed drug release that may occur with longer alkyl chains. Moreover, incorporation of PA contributes to the formation of a compact hydrophobic core that preserves the integrity of the disulfide linkage during circulation but still permits cleavage within the TME. The EM confers homologous targeting and immune evasion, promoting efficient accumulation within tumor tissues[[22], [23], [24]]. Upon cellular internalization, the intracellular reducing microenvironment cleaves the disulfide bond within DTX-SS-PA, releasing active DTX to exert cytotoxic effects. And MSA-2 is released to initiate STING-dependent immune activation. STING signaling triggered in tumor cells and transmitted to dendritic cells enhances DC maturation and activates T cells, thereby eliciting a robust antitumor immune effect (Scheme 1). Overall, the exosome-inspired nanoplatform designed for STING activation provides a potential avenue to boost both the precision and therapeutic efficacy of chemoimmunotherapy against cancer.

## 2 Materials and methods

### 2.1 Materials

PEG5k-PCL15k was purchased from Jinan DaiGang Bioengineering Co., Ltd. (Jinan, China). Glutathione (GSH), fetal bovine serum (FBS), methyl thiazolyl tetrazolium (MTT), DiI, DiR, trypsin, and culture media were sourced from Dalian Meilun Biotechnology Co., Ltd. (Dalian, China). MSA-2 came from Macklin Inc. (Shanghai, China). Hydroxybenzotriazole (HOBT) and N-(3-dimethylaminopropyl)-N'-ethylcarbodiimide hydrochloride (EDCI) were provided by Macklin Biochemical Technology Co., Ltd. (Shanghai, China). DTX, PA, 4-dimethylaminopyridine (DMAP), and coumarin-6 (C-6) were obtained from Shanghai Aladdin Biochemical Technology Co., Ltd. (Shanghai, China). Hoechst 33342, anti-CD9 antibody, anti-CD81 antibody, and the Annexin V-FITC/PI apoptosis detection kit were supplied by Beijing Solarbio Science & Technology Co., Ltd. (Beijing, China). ELISA kits were purchased from Elabscience Biotechnology Co., Ltd. (China). The Calcein AM cell viability assay kit came from Beyotime Biotechnology (Shanghai, China). Anti-CD80 and anti-CD86 antibodies were obtained from BioLegend, anti-STING antibody was bought from ABclonal (A21051), and anti-Tubulin β antibody was purchased from Bioss (bsm-33034R). Cell culture dishes, plates, and centrifuge tubes were supplied by Wuxi NEST Biotechnology Co., Ltd. (Wuxi, China). Nanoparticle preparation employed a Scientz-IID ultrasonic homogenizer (Ningbo Scientz Biotechnology Co., Ltd., China). All other reagents and solvents were of analytical grade.

### 2.2 Synthesis of DTX-SS-PA

A mixture of ethylene glycol (45.90 ml) and p-toluenesulfonic acid (0.51 g) was treated dropwise with a solution of PA (3.65 g) in toluene (2.00 ml), then heated under reflux (110 °C, 2 h), after column chromatography, the product (PA-OH) in 81% yield; 2,2′-thiodiglycolic acid (1.45 g) in acetic anhydride was stirred under N₂ for 2 h, concentrated, redissolved in dichloromethane (DCM), then treated with PA-OH (1.20 g) and DMAP (84.40 mg) in DCM at 25 °C for 12 h to afford, after column purification, the intermediate in 79.5% yield; this intermediate (69.70 mg) in DCM was mixed with EDCI (115.00 mg), HOBT (63.60 mg), and DMAP (7.35 mg), activated at 0 °C under N₂ for 2 h, reacted with DTX (100.24 mg) at 25 °C for 36 h, and purified by preparative HPLC to yield DTX-SS-PA (37%).

### 2.3 Preparation of MNPs, DNPs, MDNPs and EMMDs

MDNPs, loaded with MSA-2 and DTX-SS-PA, were prepared via emulsion solvent evaporation. A chloroform solution containing PEG-PCL, DTX-SS-PA, and MSA-2 was emulsified into water, followed by solvent evaporation and 220 nm filtration. Single drug micelles were similarly fabricated.

When 4T1 cells reached 80% confluence, exosomes were collected after 48 h of serum-free incubation. The samples were centrifuged to remove impurities, and exosome pellets were obtained by ultracentrifugation (100,000 g, 2 h). After resuspension in hypotonic buffer (containing protease inhibitors) and incubation at 4 °C for 12 h, the EM was obtained by ultracentrifugation (100,000 g, 5 h). EMMDs were fabricated by repeatedly extruding MDNPs with EM through a polycarbonate filter of 220 nm pore size (Antos Nanotechnology Co., Ltd.).

### 2.4 Characterization of MNPs, DNPs, MDNPs and EMMDs

Exosome morphology was observed using transmission electron microscopy (TEM; JEM-2100, JEOL, Japan). Western blotting was employed to verify the isolation of exosomes by detecting the marker proteins CD9 and CD81. Protein expression of CD9 and CD81 in MDNPs, EM, and EMMDs was further analyzed by western blotting after SDS-PAGE separation and Coomassie Blue staining. Morphological observation was carried out again by transmission electron microscopy. Dynamic light scattering (DLS, Malvern Instruments, UK) was used to determine hydrodynamic diameter, polydispersity index (PDI), and zeta potential. The drug contents of MSA-2 and DTX-SS-PA were determined via HPLC. To assess membrane-coating integrity, coumarin-6 (core label) and DiI (EM label) were incorporated and imaged via CLSM. EMMDs were dispersed in PBS or in culture medium with or without 10% FBS to evaluate their colloidal stability.

### 2.5 GSH-responsiveness of DTX-SS-PA and in vitro drug release

DTX was released from DTX-SS-PA and examined in PBS with 30% ethanol under shaking (100 rpm) at 37 °C using GSH concentrations of 0, 0.01 and 0.1 mM. A parallel assessment of dual drug (DTX and MSA-2) release from EMMDs was performed under consistent conditions employing a 14 kDa dialysis membrane. Samples taken at specified time points were analyzed by HPLC, with all results reported as mean ± SD (*n* = 3).

### 2.6 Cell culture

The 4T1 and B16F10 cell lines were cultured separately in RPMI-1640 and high-glucose DMEM (supplemented with 10% FBS and 100 µg/ml penicillin/streptomycin). Subculturing was performed using trypsin at ∼80% confluency, with medium changes every 2–3 d.

### 2.7 Cellular uptake

4T1 cells (2.0 × 10⁴/well) were seeded in 24-well plates and cultured for 24 h. The cells were then treated with coumarin-6-labeled PEG-PCL nanoparticles (C-6 NPs) or EM-coated PEG-PCL nanoparticles (EM@C-6 NPs) at a concentration of 250 ng/ml for 0.5 or 2 h. After rinsing with PBS, cells were fixed with 4% paraformaldehyde (PFA) and stained with Hoechst 33342. Fluorescence images were taken by confocal laser scanning microscopy (CLSM, C2SI, Nikon, Japan), and flow cytometry (Becton, Dickinson and Company, USA) was used for quantification.

### 2.8 Cytotoxicity assay

4T1 and B16F10 cells (2000 cells/well) were plated in 96-well plates and allowed to attach overnight. After exposure to different concentrations of MNPs, DNPs, MDNPs, or EMMDs for 48 h, MTT solution was added and incubated for 4 h. DMSO was then added to measure absorbance.

### 2.9 Cell apoptosis

After overnight incubation, 4T1 cells (2.0 × 10⁵/well in 12-well plates) were exposed to different formulations (PBS, MNPs, DNPs, MDNPs and EMMDs) for 24 h. The treated cells were then collected, stained with Annexin V-FITC/PI, and analyzed by flow cytometry to assess apoptosis.

### 2.10 Live/Dead cell staining

4T1 cells were grown on glass coverslips in 24-well plates overnight, followed by treatment with PBS, MNPs, DNPs, MDNPs, or EMMDs for 24 h. After removing the medium, cells were rinsed twice with PBS and stained with the Calcein AM cell viability assay kit following the manufacturer’s protocol. Live/dead staining images were captured by CLSM.

### 2.11 STING pathway activation in cancer cells and BMDCs

4T1 cells were seeded in 6-well plates and treated with PBS, MNPs, DNPs, MDNPs or EMMDs. After incubation, supernatants were collected for ELISA to detect IFN-γ, IL-6 and TNF-α, reflecting STING pathway activation. Bone marrow-derived dendritic cells (BMDCs) were isolated from BALB/c mice and co-cultured with the pretreated 4T1 cells for 24 h. The resulting supernatants were analyzed by ELISA for cytokine secretion, and non-adherent BMDCs were collected for flow cytometry.

### 2.12 Animal studies

All animal experiments were performed in accordance with the ethical guidelines approved by the Institutional Animal Ethics Committee of Shenyang Pharmaceutical University.

### 2.13 Biodistribution

A breast tumor model was established by subcutaneous injection of 5 × 10⁶ 4T1 cells into the right flank of female BALB/c mice (6 weeks old, ∼20 g). When tumors reached ∼300 mm³, DiR NPs or EM@DiR NPs were injected intravenously at 1 mg/kg (DiR equivalent). Fluorescence imaging was conducted at 1, 2, 4, 8, 12 and 24 h using an IVIS system. After 24 h, mice were euthanized, and tumors and major organs were collected for *ex vivo* imaging.

### 2.14 In vivo antitumor efficacy

BALB/c mice (6 weeks old) were subcutaneously inoculated with 4T1 cells (5 × 10⁶) in the right flank to establish a tumor model. When tumors reached ∼100 mm³, the mice were randomized (*n* = 5) and injected intravenously . Treatments were repeated every 72 h. Tumor size and body weight were tracked daily until Day 15, when mice were euthanized. Tumors were excised, photographed, and weighed; their volume was calculated as (length × width²)/2, and tumor burden was expressed as the ratio of tumor to body weight. Blood was collected and centrifuged (3500 rpm, 10 min) for plasma biochemical evaluation (ALT, AST, BUN, CREA). Major organs together with tumor tissues were fixed, sectioned, and examined by hematoxylin and eosin (H&E) staining for histopathological assessment. Apoptosis and proliferation were further determined using terminal deoxynucleotidyl transferase dUTP nick-end labeling (TUNEL) and Ki67 staining.

### 2.15 Western blotting assay

Proteins from 4T1 tumor lysates were quantified by the BCA assay, separated by SDS-PAGE, and transferred to PVDF membranes. After blocking, membranes were incubated with primary antibodies against IRF3, TBK1, STING and β-tubulin, followed by HRP-conjugated secondary antibody. Signals were detected using an ECL system.

### 2.16 In vivo antitumor immune responses

To evaluate DC maturation *in vivo*, lymph nodes (LNs) were gently ground through a 200-mesh sieve. Single cell suspensions were labeled with FITC-anti-CD80 and PE-anti-CD86 antibodies, followed by flow cytometric analysis using a CD11c^+^ cell sorting. CD8^+^ T cell proportions were determined with a CD3^+^ cell sorting following staining with FITC-anti-CD3 and PE-anti-CD8 antibodies. Regulatory T cell (Treg) frequencies were assessed using a CD4^+^ cell sorting to evaluate immunosuppressive activity.

For tumoral DC analysis, dissociated tumor cells were stained and mature DCs were quantified via CD11c^+^ cell sorting with flow cytometry. T cell infiltration was examined using a CD3^+^ cell sorting after PE-anti-CD8 antibody staining. Treg ratios were determined with a CD4^+^ cell sorting to assess immunosuppression. Quantification of cytokine levels (IFN-β, IFN-γ, IL-6 and TNF-α) in tumor tissues was performed using ELISA kits according to manufacturer protocols.

### 2.17 Statistical analysis

Data are expressed as mean ± SD. Statistical analyses were performed using a two-tailed Student’s t-test or one-way ANOVA. A value of *P* < 0.05 was considered statistically significant. *P < 0.05, **P < 0.01, ***P < 0.001, ****P < 0.0001.

## 3 Results and discussion

### 3.1 Preparation and characterization of the nanosystem

**Fig. 1:**
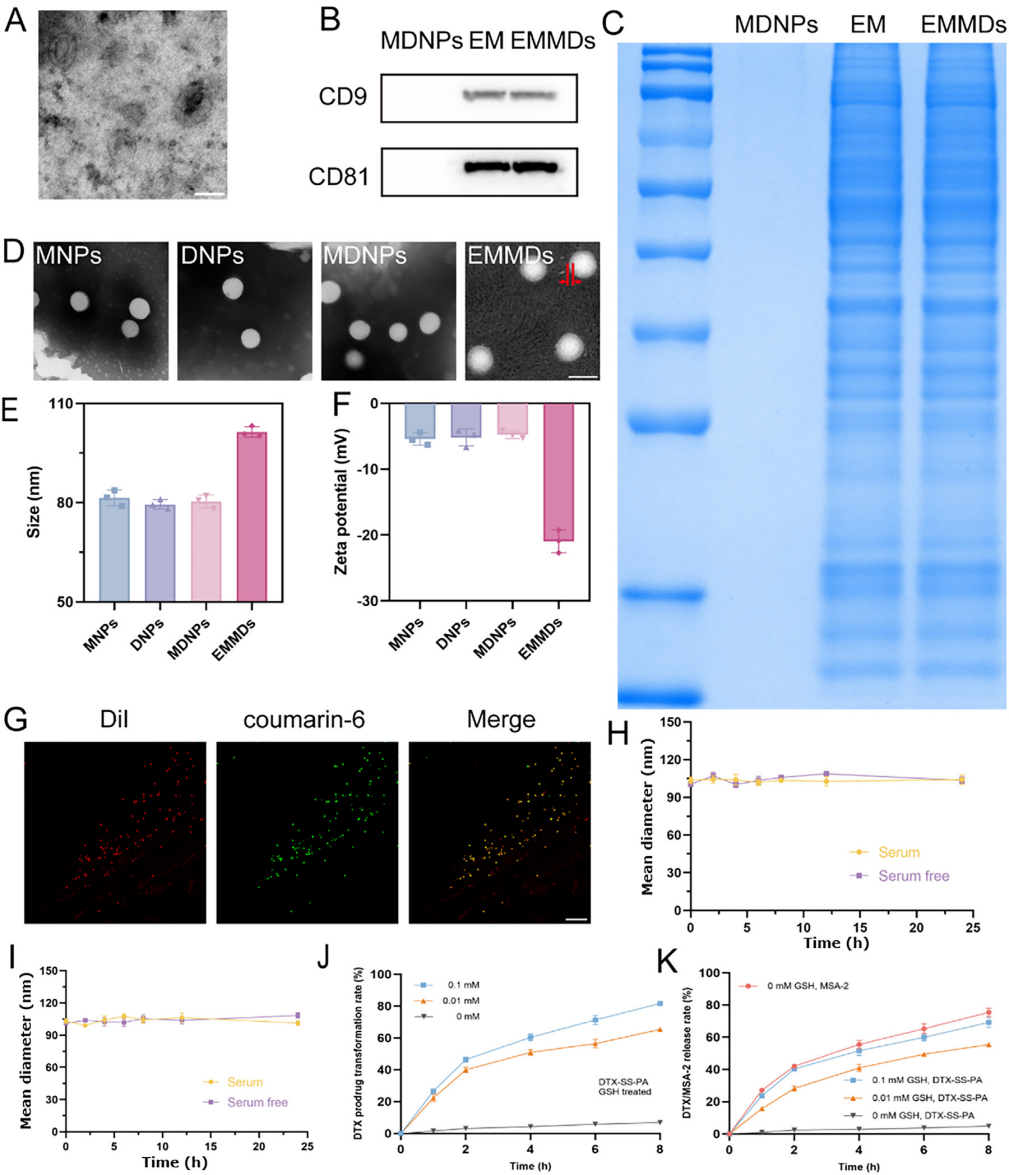
(A) Exosomes derived from 4T1 cells were characterized by TEM imaging. Scale bar: 100 nm; (B) Immunodetection of CD9 and CD81 in MDNPs, EM, and EMMDs; (C) Protein profiles of MDNPs, EM, and EMMDs were assessed by SDS-PAGE; (D) Representative TEM micrographs display the morphology and size of MNPs, DNPs, MDNPs and EMMDs. Scale bar: 100 nm; (E) Hydrodynamic size and (F) zeta potential values were determined for MNPs, DNPs, MDNPs and EMMDs; (G) Fluorescence imaging revealed the co-localization of the EM shell (red) with the PEG-PCL core (green). Scale bar: 1 µm; Stability of EMMDs in colloidal form was analyzed in (H) PBS (pH 7.4) and (I) culture medium either containing or without 10% FBS (*n* = 3); (J) Reduction-responsive conversion of DTX-SS-PA to DTX triggered by GSH was depicted (*n* = 3); (K) Cumulative release profiles of MSA-2 and DTX from EMMDs were evaluated at different GSH concentrations *in vitro* (*n* = 3).

First, DTX was conjugated with PA via a disulfide bond to synthesize the prodrug DTX-SS-PA (Scheme S1). Its chemical structure was confirmed by mass spectrometry (MS) and ^1^H NMR spectroscopy. HPLC analysis showed that the purity of DTX-SS-PA reached 99.98%, indicating that it was suitable for subsequent experiments (Fig. S1). Using the emulsion-solvent evaporation method, MSA-2 and DTX-SS-PA were co-encapsulated in PEG-PCL nanoparticles to prepare MDNPs, where “M” represents MSA-2 and “D” represents DTX-SS-PA [[25], [26], [27]]. As a control, MSA-2 and DTX-SS-PA were separately loaded under the same conditions to prepare MNPs and DNPs. The exosomes were isolated by density gradient ultracentrifugation, and then the EM was extracted by a hypotonic lysis method. Subsequently, MDNPs were co-incubated with the EM to form EMMDs [28]. The typical cup-shaped morphology of exosomes was observed through TEM, and the marker proteins CD9 and CD81 of the exosomes were detected by Western blot, thus confirming the identity of the exosomes [29] (Figs. 1A and S2). Further SDS-PAGE and Western blot analyses confirmed that EMMDs after membrane coating still retained the characteristic protein profile of native exosomes [30] (Fig. 1Band 1C). In addition, the hydrodynamic diameter and morphological characteristics of MNPs, DNPs, and MDNPs indicated that these nanoparticles exhibited a uniform spherical morphology with an average particle size of about 80 nm (Fig. 1D). The successful encapsulation of PEG-PCL nanoparticles by the EM was confirmed through a decrease in zeta potential, an increase in hydrodynamic size to about 100 nm, and TEM observations providing direct morphological evidence (Fig. 1Eand 1F).

The encapsulation efficiencies of MSA-2 and DTX-SS-PA in PEG-PCL nanoparticles were determined to be 74.2% and 91%, respectively. Dual-fluorescence labeling with DiI (membrane, red) and coumarin-6 (core, green) enabled CLSM evaluation of the structural integrity of bioinspired nanoparticles [31]. As shown in Fig. 1G, strong colocalization of red and green fluorescence confirmed the successful fabrication of the exosome-mimetic nanoparticles. Furthermore, EMMDs exhibited high colloidal stability over 24 h in PBS and cell culture media, regardless of the presence of 10% FBS (Fig. 1Hand 1I).

### 3.2 Reducibility of DTX-SS-PA and in vitro drug release

The therapeutic performance and biosafety of DTX are closely dependent on its release behavior from PEG-PCL nanoparticles. We have previously shown that disulfide bonds confer reduction responsiveness and elucidated the associated release mechanism. Herein, GSH was utilized as a reductant to assess the reduction-triggered release behavior of DTX-SS-PA [32]. As illustrated in Fig. 1J, the cumulative release of DTX from DTX-SS-PA stayed under 10% after 12 h in the absence of GSH, indicating minimal leakage under physiological conditions and demonstrating its stability in normal tissues, which helps reduce off-target toxicity. Under the action of 0.01 mM and 0.1 mM GSH, DTX-SS-PA rapidly released DTX, reaching approximately 60% within 8 h and an overall release of about 80%, demonstrating good reduction-responsive characteristics of the prodrug.

The release behavior of EMMDs under different concentrations of GSH was further studied, and the two preparations showed distinct release characteristics. In the absence of GSH, the release rate of MSA-2 within 12 h was significantly faster than that of DTX-SS-PA, suggesting the presence of a mechanism independent of GSH concentration that can rapidly promote immunomodulation. In contrast, the release of DTX from DTX-SS-PA obviously depended on the concentration of GSH. Under 0.1 mM GSH, the release within 12 h reached approximately 70% (Fig. 1K). Disulfide bonds can be specifically cleaved by GSH in cells, enabling drug activation in the TME. This feature supports the synergistic antitumor effect of combined immune activation and chemotherapy.

### 3.3 Cellular uptake

**Fig. 2:**
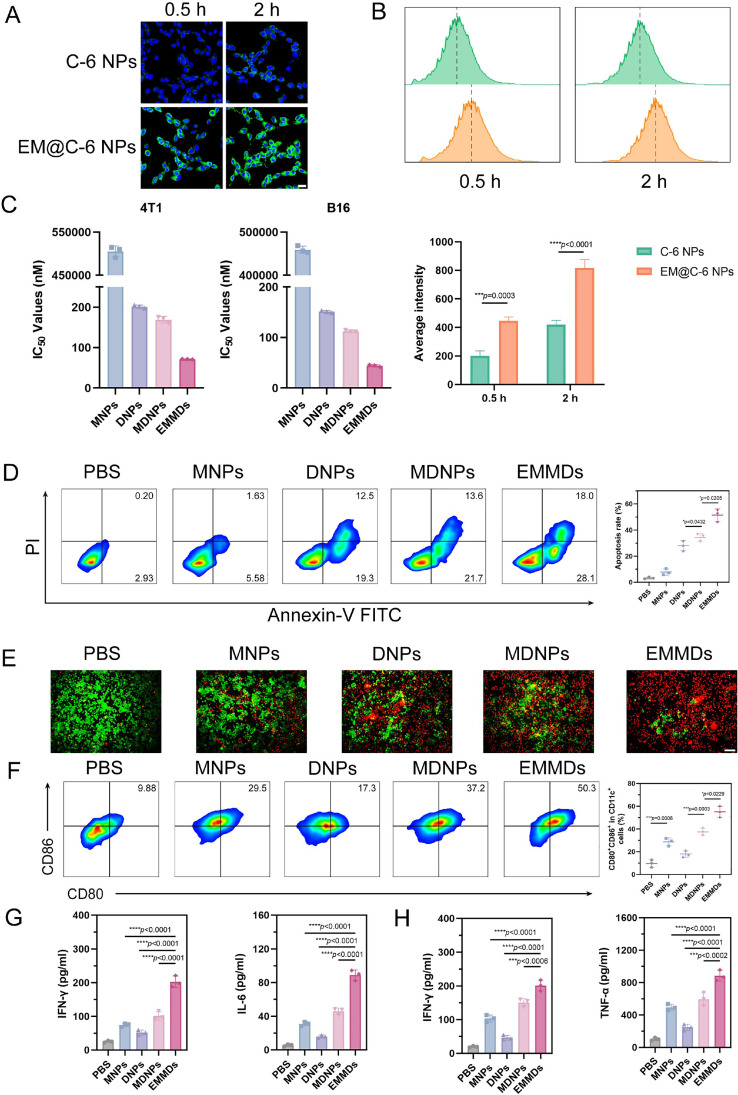
(A) Cellular uptake was visualized via fluorescence imaging and (B) quantified by flow cytometry following incubation of 4T1 cells with C-6 NPs and EM@C-6 NPs for 0.5 and 2 h (*n* = 3). Scale bar: 20 µm; (C) IC_50_ values of MNPs, DNPs, MDNPs, and EMMDs in 4T1 cells and B16F10 cells (*n* = 3); (D) Apoptosis of 4T1 cells after treatment with MNPs, DNPs, MDNPs, or EMMDs (*n* = 3); (E) Viability of 4T1 cells after treatment with MNPs, DNPs, MDNPs and EMMDs was assessed using live/dead staining (*n* = 3). Scale bar: 100 µm; (F) Expression of DC maturation markers CD80 and CD86 analyzed by flow cytometry (*n* = 3); (G) IFN-γ and IL-6 levels in 4T1 cells post-treatment were quantified (*n* = 3); (H) Concentrations of IFN-γ and TNF-α in DCs incubation with treated cancer cells (*n* = 3). Data are presented as mean ± SD. Statistical significance was determined by a two-tailed Student’s *t*-test for panel B, and by one-way ANOVA with Tukey’s test for panels D, F, G, and H, *P < 0.05, **P < 0.01, ***P < 0.001, ****P < 0.0001, ns indicates not significant.

EMMDs exhibited good antitumor potential, mainly due to their efficient cellular uptake. To evaluate this property, the uptake of C-6 NPs and EM@C-6 NPs in 4T1 cells was compared and analyzed. Confocal microscopy showed that intracellular fluorescence increased over time, and the signal at 2 h was significantly higher than that at 0.5 h (Fig. 2A). In addition, at the same time point, the fluorescence intensity of EM@C-6 NPs was significantly higher than that of C-6 NPs, indicating that proteins on the EM promoted cellular uptake. Flow cytometry analysis demonstrated that after 0.5 h and 2 h of incubation, the fluorescence intensity of 4T1 cells treated with EM@C-6 NPs was approximately 2.2-fold and 1.9-fold higher than that of cells treated with C-6 NPs, respectively (Fig. 2B). These results indicated that the EM significantly enhanced nanoparticle uptake by tumor cells.

### 3.4 In vitro antitumor activity

Compared with normal tissue, tumor tissue has a unique intracellular redox environment, which provides a theoretical basis for the construction of a redox-responsive prodrug nanodelivery system that enables drugs to be selectively released and improves the safety of chemotherapy. Based on this, we used MTT colorimetry to evaluate the cytotoxicity of four preparations (MNPs, DNPs, MDNPs and EMMDs) against 4T1 and B16F10 cell lines. The results showed that MSA-2 alone had almost no cytotoxicity, which was consistent with its role as an immunomodulator rather than a drug that directly induces cytotoxicity in cells. Among the four preparations, EMMDs were the most cytotoxic, followed by MDNPs, DNPs, and MNPs (Figs. 2C and S3). The good cellular uptake ability of EMMDs seemed to be an important reason for the enhancement of their therapeutic effect. The experimental results reflected the combined effect of prodrug activation and intracellular synergy.

Subsequently, the apoptosis induced by different preparations in tumor cells was evaluated. The apoptosis rate of the blank control group was 3.13%, while the apoptosis rates of EMMDs, MDNPs, DNPs, and MNPs groups increased to 46.1%, 35.3%, 31.8% and 7.21%, respectively, which was consistent with the cytotoxicity results described above (Fig. 2D). The use of calcein AM for live-cell imaging further confirmed the apoptosis-promoting effect of EMMDs, which was consistent with the quantitative analysis results (Fig. 2E). These results demonstrated that the synergy of targeted delivery, efficient cellular uptake, and prodrug activation enabled EMMDs to induce significant antitumor activity at the cellular level.

### 3.5 STING pathway activation

In order to evaluate the maturation of DCs, 4T1 cells treated with different preparations were harvested and cultured with BMDCs. The results showed that the proportion of mature DCs increased significantly, among which EMMDs, MDNPs, and MNPs groups reached 50.3%, 37.2% and 29.5%, respectively. In contrast, the DC maturation rate of DNPs treatment group was only 17.3%. Experimental results showed that EMMDs could activate DCs more effectively, reflecting the enhanced immune activation effect of nanosystems co-delivering chemotherapy prodrugs and STING agonists (Fig. 2F).

As a STING agonist encapsulated in EMMDs, MSA-2 aimed to amplify the antitumor immune response. Furthermore, by analyzing the relevant signaling pathway, the activation of the STING pathway was investigated. EMMDs significantly activated the STING signaling pathway, which was characterized by an increase in the secretion levels of IFN-γ and IL-6 in 4T1 cells, as well as elevated levels of IFN-γ and TNF-α in DCs (Fig. 2Gand 2H). The above results showed that EMMDs could not only trigger the activation of the STING signaling pathway, but also promote DC maturation and antigen presentation.

### 3.6 Biodistribution

**Fig. 3:**
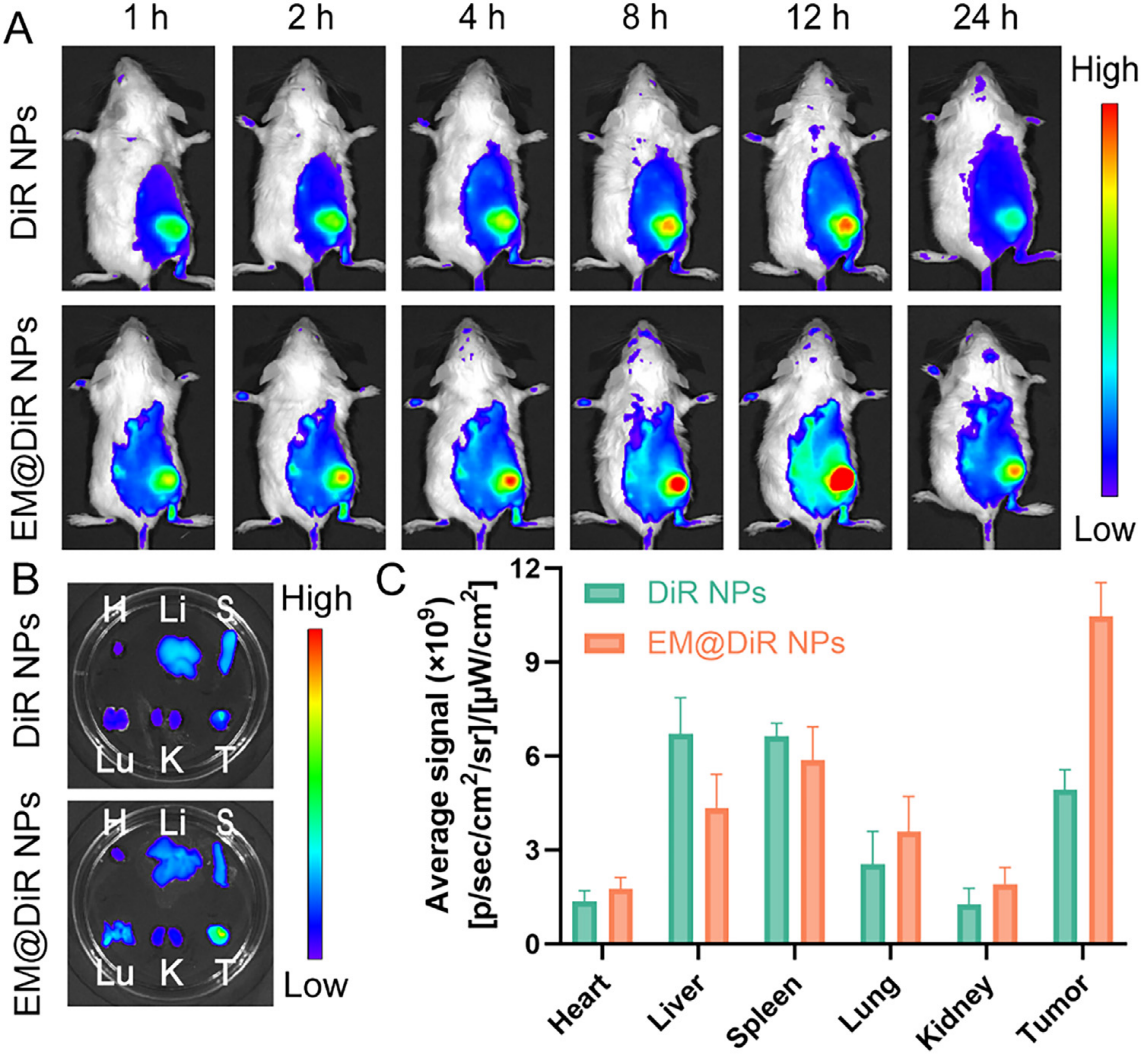
(A) *In vivo* fluorescence was tracked at 1, 2, 4, 8, 12 and 24 h after administering with DiR NPs and EM@DiR NPs to BALB/c mice bearing 4T1 tumors (*n* = 3); (B) *Ex vivo* fluorescence imaging of excised tissues (*n* = 3); (C) ROI analysis of major organs and tumors collected 24 h post-injection (*n* = 3).

To evaluate the tumor-targeting ability of exosome-mimetic nanoparticles, a 4T1 xenograft tumor model was established. Fluorescence imaging results showed that the signal intensity of EM@DiR NPs continued to increase from 1 to 12 h and was always higher than that of the DiR NPs group (Fig. 3A). At 24 h, *ex vivo* tumor imaging revealed significantly stronger retention of EM@DiR NPs, approximately 2.12-fold higher than the control (Fig. 3Band 3C). This enhanced enrichment effect may be due to homotypic targeting mediated by EM proteins, and the bionic coating prolonged circulation time. Experimental data showed that the self-recognition mechanism mediated by membrane proteins promoted the active uptake of tumor cells, while the inherent immune-escape characteristics of the EM helped to prolong blood circulation and improve drug delivery at the tumor site.

### 3.7 In vivo antitumor efficacy

**Fig. 4:**
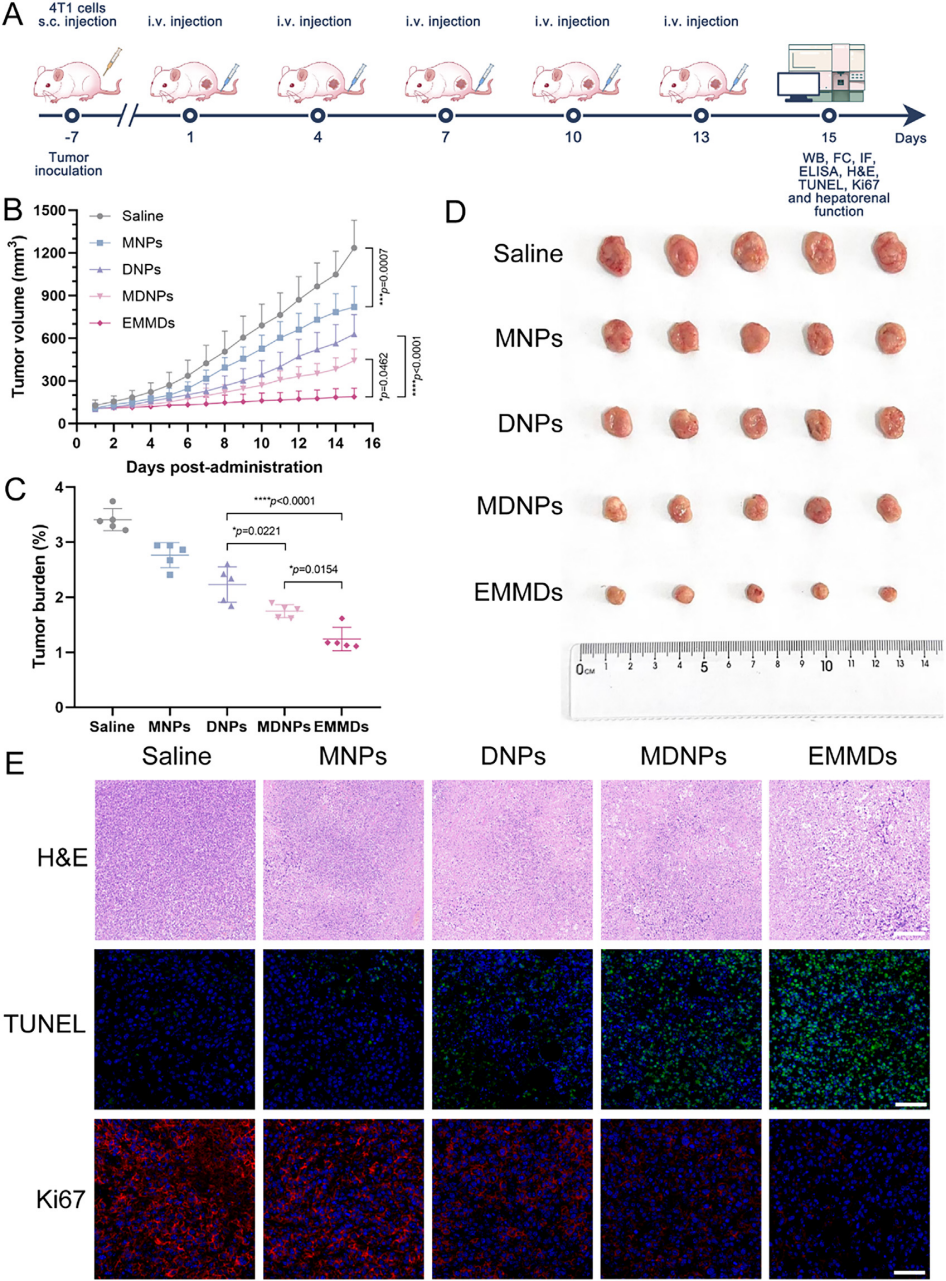
(A) 4T1 xenograft tumor treatment; (B) Tumor growth curves in 4T1 tumor model following treatments with different formulations (*n* = 5); (C) Tumor burden at the end of the study (*n* = 5); (D) Photographs of excised tumors from each group after the final treatment (*n* = 5); (E) H&E, TUNEL, and Ki67 staining of tumors from treated 4T1 tumor-bearing mice (*n* = 5). Scale bar: 100 µm. Data are presented as mean ± SD. Statistical significance was determined by one-way ANOVA with Tukey’s test, *P < 0.05, **P < 0.01, ***P < 0.001, ****P < 0.0001, ns indicates not significant.

The antitumor effects of exosome-mimetic microparticles (EMMDs) were examined in a murine 4T1 subcutaneous tumor model (Fig. 4A). Tumors in the control group grew rapidly, reaching approximately 1200 mm^3^ by Day 15. The groups treated with different preparations showed varying degrees of tumor growth inhibition. Among them, the final tumor volumes of MNPs, DNPs, and MDNPs groups were approximately 800 mm^3^, 600 mm^3^ and 400 mm^3^, respectively. EMMDs showed the strongest antitumor effect, with tumor volume maintained at about 200 mm³ on Day 15, significantly lower than that of all other treatment groups (Fig. 4B–4D). No obvious systemic toxicity was observed in mice treated with EMMDs; their body weight remained stable, and liver and kidney function indicators were within the normal range (Fig. S4 and S5). H&E staining of major organs further confirmed the biosafety of the preparation, with no obvious pathological changes (Fig. S6). The enhanced antitumor activity of EMMDs was also reflected by increased tumor cell apoptosis (TUNEL) and decreased proliferation (Ki67) (Fig. 4E). Compared with MNPs, DNPs and MDNPs, the superior antitumor efficacy of EMMDs could be attributed to its bionic EM, which promoted homotypic tumor targeting, extended circulation time, and enabled selective tumor enrichment, thus contributing to its outstanding therapeutic efficacy.

### 3.8 In vivo antitumor immune responses

**Fig. 5:**
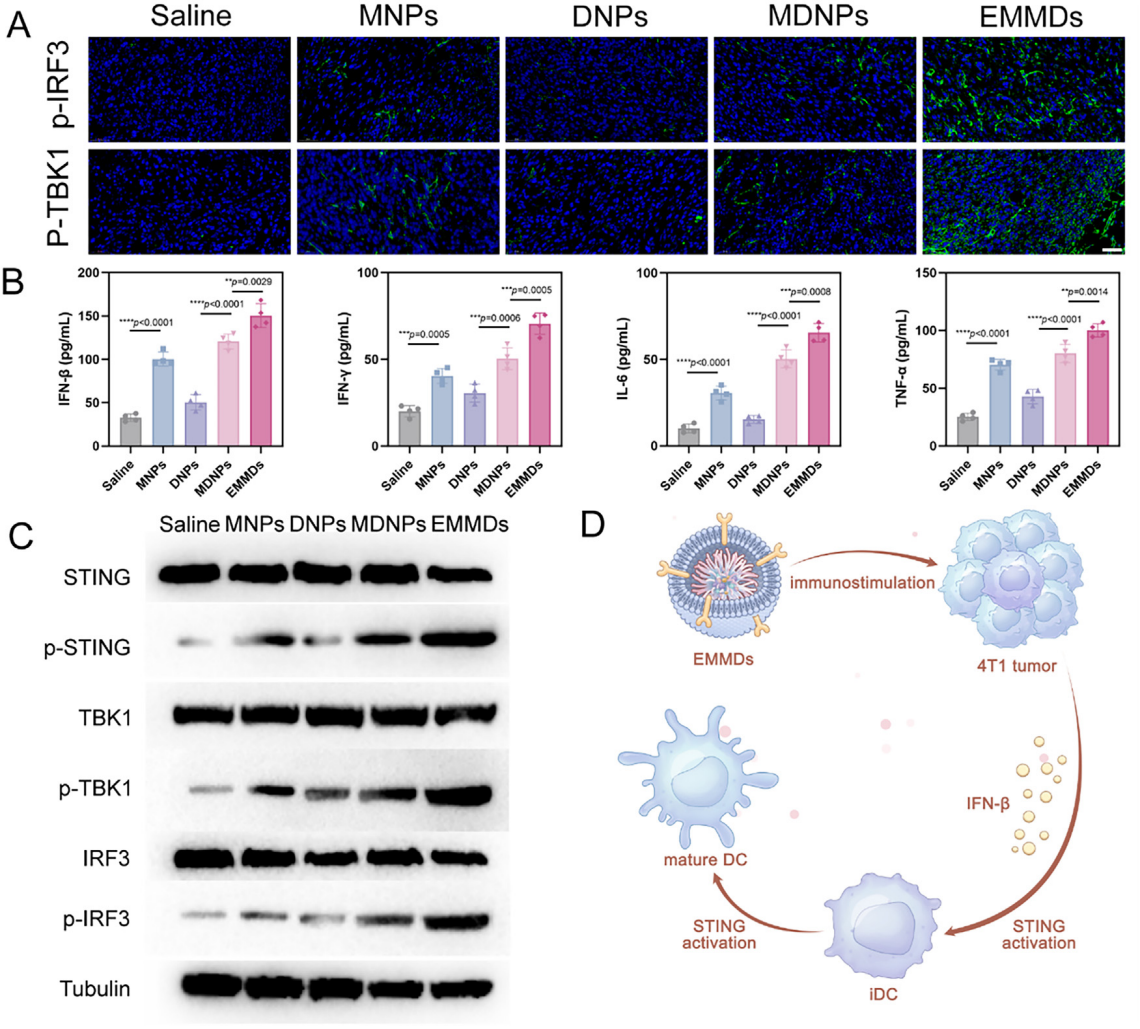
(A) Immunofluorescence staining of p-TBK1 and p-IRF3 in tumor tissues across treatment groups (*n* = 4). Scale bar: 50 µm; (B) IFN-β, IFN-γ, IL-6 and TNF-α in tumor tissues were measured by ELISA (*n* = 4); (C) Western blotting analysis assessed the phosphorylation of STING, TBK1, and IRF-3 in tumor tissues; (D) EMMDs-induced STING activation in the TME. Data are presented as mean ± SD. Statistical significance was determined by one-way ANOVA with Tukey’s test, *P < 0.05, **P < 0.01, ***P < 0.001, ****P < 0.0001, ns indicates not significant.

EMMDs-driven immune activation was further assessed via phenotypic analysis of immune cell populations within the TME and LNs. Compared to control groups (Saline, DNPs, MNPs and MDNPs), EMMDs treatment resulted in strongly upregulated p-TBK1 and p-IRF3 expression, as evidenced by immunofluorescence staining of tumor sections, indicating potent STING pathway activation (Fig. 5A). ELISA and reverse Transcription quantitative Polymerase Chain Reaction (RT-qPCR) confirmed enhanced secretion of STING-dependent cytokines (IFN-β, IFN-γ, IL-6 and TNF-α), demonstrating robust pathway activation (Figs. 5B and S7). As shown in Fig. 5C, Western blot analysis assessed the phosphorylation of STING, TBK1 and IRF-3 in tumor tissues. EMMDs induced STING activation in TME (Fig. 5D).

**Fig. 6:**
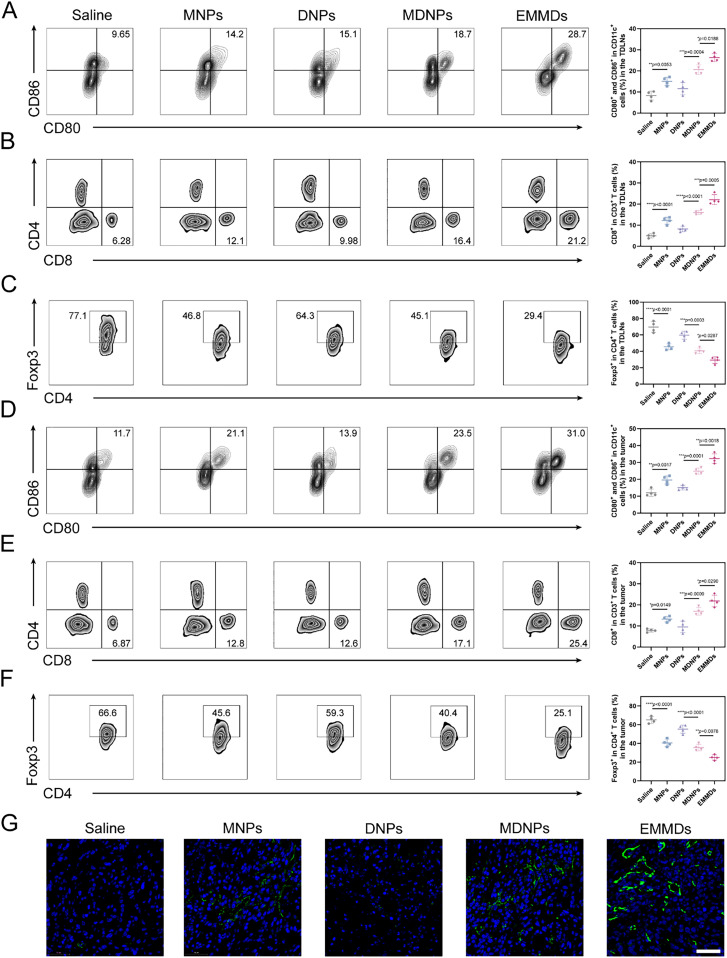
(A-C) Flow cytometric analysis of immune cell populations in tumor-draining LNs, including (A) mature dendritic cells (CD11c⁺CD80⁺CD86⁺), (B) CD8⁺ T cells among CD3⁺ T cells, and (C) CD4^+^Foxp3^+^ Tregs (*n* = 4); (D-F) Flow cytometric analysis of immune cell populations in tumor tissues, including (D) mature dendritic cells (CD11c⁺CD80⁺CD86⁺), (E) CD8⁺ T cells among CD3⁺ T cells, and (F) CD4^+^Foxp3^+^ Tregs (*n* = 4); (G) Immunofluorescence images of CD8⁺ T cells in tumor tissues. Scale bar: 50 µm. Data are presented as mean ± SD. Statistical significance was determined by one-way ANOVA with Tukey’s test, *P < 0.05, **P < 0.01, ***P < 0.001, ****P < 0.0001, ns indicates not significant.

Flow cytometric results indicated that EMMDs elicited the strongest enhancement in antitumor immune response. The proportion of mature dendritic cells (CD80^+^CD86^+^) induced by EMMDs in LNs was the highest, significantly higher than that of MDNPs, MNPs, and DNPs. At the same time, the activation of CD8^+^T cells was significantly enhanced (Fig. 6Aand 6B), while the proportion of regulatory T cells (Treg) was significantly reduced (Fig. 6C). In tumor tissue, EMMDs were also most effective in promoting DC maturation, significantly increasing the proportion of tumor-infiltrating CD8^+^ T cells, and reducing the Treg population (Fig. 6D–6G). These results showed that EMMDs could stimulate strong and specific antitumor immune responses in the TME while relieving immunosuppression.

Through the targeted delivery of MSA-2 by EMMDs, the STING pathway in the tumor was strongly activated. This activation promoted the production of type I interferons and pro-inflammatory cytokines, reshaped the TME, and enhanced the systemic antitumor immune response. The enhancement of the efficacy of EMMDs mainly depended on its bionic EM, which achieved homotypic tumor targeting and promoted tumor-specific enrichment. Subsequently, the controlled release of MSA-2 in the cell activated the STING pathway, enhancing DC maturation and antigen cross-presentation, thus promoting the activation and tumor infiltration of a large number of cytotoxic CD8^+^ T cells. At the same time, EMMDs inhibited Treg activity and weakened immunosuppression. Overall, precise tumor-targeted delivery and immune regulation jointly supported the strong antitumor performance observed by EMMDs in the 4T1 mouse model.

## 4 Conclusions

This study presents a biomimetic co-delivery strategy designed to overcome immunosuppression in TNBC. The nanosystem, coated with EM derived from 4T1 cancer cells, enhances tumor-targeting specificity and addresses key barriers in drug delivery. Compared with uncoated nanoparticles, EMMDs exhibited improved cellular uptake and tumor accumulation, consistent with homotypic targeting mediated by tumor cell-derived EM. At the tumor site, the DTX prodrug was selectively activated to induce cytotoxic effects, while MSA-2 was released to engage the STING pathway. Evidence of TBK1/IRF3 phosphorylation and elevated cytokine levels confirmed STING activation and its contribution to antitumor immune responses. This triggered a type I interferon response that reshaped the immunosuppressive TME and initiated sustained immune activity. By integrating chemotherapy with STING-mediated immunotherapy, EMMDs achieve synergistic antitumor effects in the 4T1 model. Overall, this work highlights a biomimetic strategy that combines targeted drug delivery with immune stimulation to enhance therapeutic efficacy. Future developments of such nanoplatforms may focus on enhancing reproducibility, controlling drug release, and better understanding interactions with the TME, while maintaining the combined chemo- and immunotherapeutic efficacy safely.

## CRediT authorship contribution statement

**Xinying Wang:** Writing – review & editing, Writing – original draft, Visualization, Validation, Supervision, Software, Resources, Project administration, Methodology, Investigation, Formal analysis, Data curation, Conceptualization. **Xianlu Zhang:** Writing – review & editing, Methodology, Data curation. **Zixuan Jiao:** Writing – review & editing, Visualization, Formal analysis, Data curation. **Shipeng Ning:** Writing – review & editing, Visualization, Validation, Software, Resources, Methodology, Formal analysis, Data curation. **Hanping Wang:** Writing – review & editing, Visualization, Formal analysis, Data curation. **Hui Liu:** Writing – review & editing, Resources, Methodology. **Lifen He:** Writing – review & editing, Resources, Methodology. **Haonan Li:** Writing – review & editing, Resources, Methodology. **Mingzhong Li:** Writing – review & editing, Writing – original draft, Supervision, Conceptualization. **Kaiyuan Wang:** Writing – review & editing, Writing – original draft, Supervision, Conceptualization. **You Pan:** Writing – review & editing, Supervision, Conceptualization. **Zhonggui He:** Writing – review & editing, Supervision, Conceptualization.

## Conflicts of interest

Authors declare that they have no competing interests.

## Appendix Supplementary materials

Image, application 1
