# Supplementary material for: An exosome-inspired docetaxel prodrug nanoplatform for potent STING activation and synergistic chemoimmunotherapy
Source: Asian J Pharm Sci. 2026 Feb 27;21(2):101139. doi: 10.1016/j.ajps.2026.101139 (PMC13089015; doi:10.1016/j.ajps.2026.101139)
Supplement: Supplementary file 1 [file mmc1.docx]

**Supplementary Information**

**An Exosome-Inspired Docetaxel Prodrug Nanoplatform for Potent STING Activation and Synergistic Chemoimmunotherapy**

Xinying Wang^a, 1^, Xianlu Zhang^f, 1^, Zixuan Jiao^a^, Shipeng Ning^c^, Hanping Wang^a^, Hui Liu^c^, Lifen He^c^, Haonan Li^g^, Mingzhong Li^b, e,*^, Kaiyuan Wang^a, b, d,*^, Zhonggui He^a, b,*^, You Pan^c,*^

^a^Department of Pharmaceutics, Wuya College of Innovation, Shenyang Pharmaceutical University, Shenyang 110016, China

^b^Joint International Research Laboratory of Intelligent Drug Delivery Systems of Ministry of Education, Shenyang Pharmaceutical University, Shenyang 110016, China

^c^Department of Breast Surgery, The Second Affiliated Hospital of Guangxi Medical University, Nanning 530000, China

^d^Departments of Diagnostic Radiology, Surgery, Chemical and Biomolecular Engineering, and Biomedical Engineering, Yong Loo Lin School of Medicine and College of Design and Engineering, National University of Singapore, Singapore 119074, Singapore

^e^School of Pharmacy, De Montfort University, The Gateway, Leicester, LE1 9BH, UK

^f^Department of Urology, the First Hospital of China Medical University, 155 North Nanjing Street, Shenyang 110000, China

^g^Hainan Branch, Shanghai Children’s Medical Center, School of Medicine, Shanghai Jiao Tong University, Sanya, China

^1^These authors contributed equally to this work.

^*^Corresponding authors.

**Supplementary Figures**





**Scheme S1.** Synthetic routes of DTX-SS-PA.


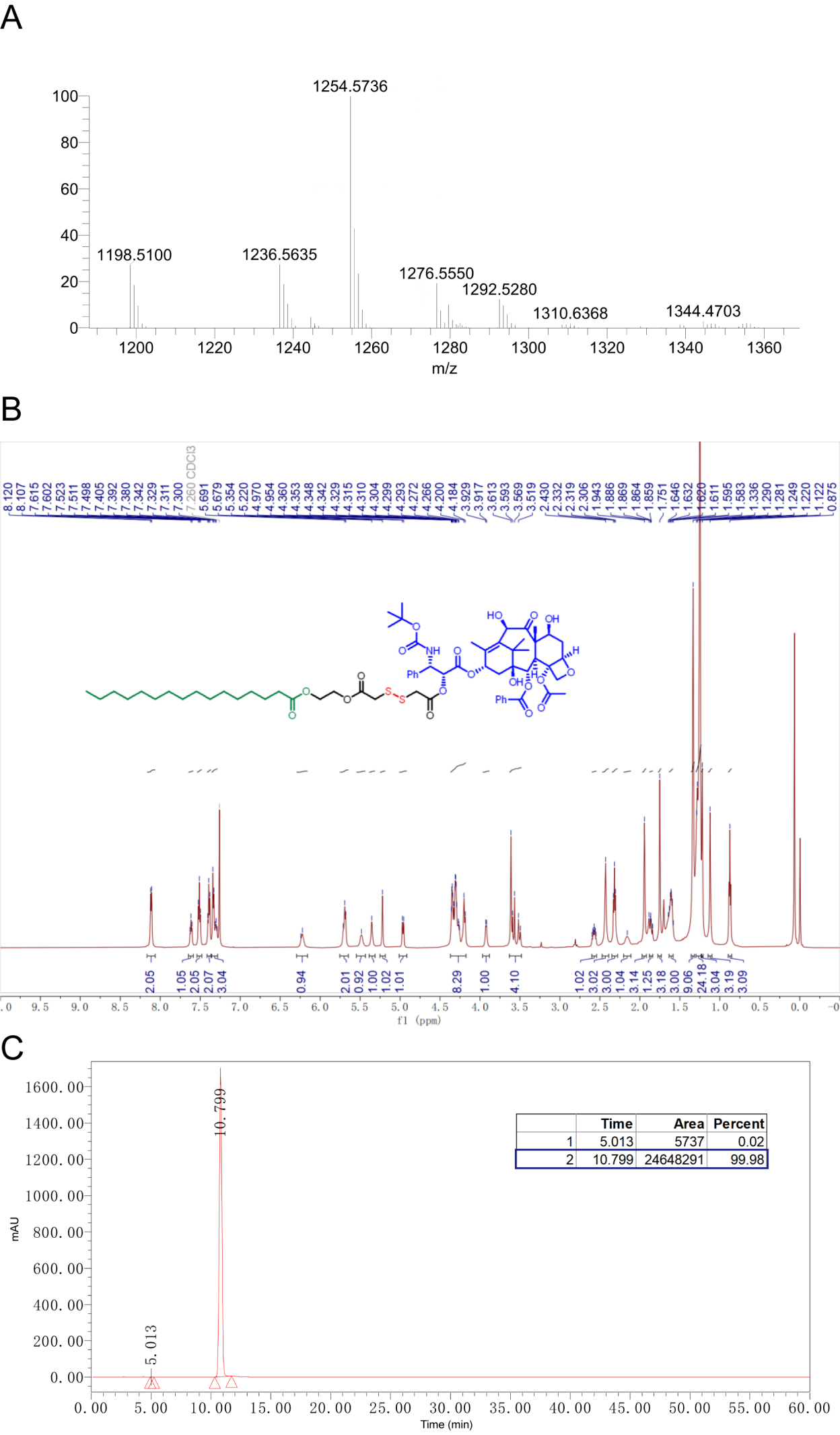


**Figure S1.** Characterizations of DTX-SS-PA. (A) Mass spectrum. (B) ^1^H NMR spectrum in CDCl_3_. (C) The purity.

ESI-MS (m/z): called for [C_65_H91NO19S_2_] [M+H]^+^ = 1254.56; found: 1254.5736.

^1^H NMR (400 MHz, Chloroform-*d*) δ 8.11 (d, *J* = 7.7 Hz, 2H, Ar-H), 7.61 (t, *J* = 7.3 Hz, 1H, Ar-H), 7.51 (t, *J* = 7.6 Hz, 2H, Ar-H), 7.39 (t, *J* = 7.5 Hz, 2H, Ar-H), 7.36 - 7.28 (m, 3H, Ar-H), 6.21 (s, 1H, 2’-H), 5.75 - 5.60 (m, 2H, 3’-H, -OH), 5.48 (s, 1H, 13-H), 5.35 (s, 1H, -OH), 5.22 (s, 1H, 10-H), 4.96 (dd, *J* = 9.6, 2.3 Hz, 1H, 2-H), 4.37 – 4.18 (m, 8H, 20-CH_2_, 5-H, -OCH_2_CH_2_O-, -OH), 3.92 (d, *J* = 6.9 Hz, 1H, 7-H), 3.63 – 3.48 (m, 4H, -CH_2_SSCH_2_-), 2.57 (ddd, *J* = 15.5, 9.5, 6.3 Hz, 1H, 3-H), 2.43 (s, 3H, -OCOCH_3_), 2.35 - 2.28 (m, 3H, -COCH_2_-, 14α-CH_2_), 2.24 - 2.12 (m, 1H, 14β-CH_2_), 1.94 (s, 3H, 18-CH_3_), 1.88 – 1.84 (m, 1H, 6α-CH_2_), 1.75 (s, 3H, 19-CH_3_), 1.63 - 1.57 (m, 3H, -OCOCH_2_CH_2_-, 6β-CH_2_), 1.34 (s, 9H, -C(CH_3_)_3_), 1.30 - 1.24 (m, 24H, -(CH_2_)_12_), 1.22 (s, 3H, 16-CH_3_), 1.12 (s, 3H, 17-CH_3_), 0.88 (d, *J* = 6.3 Hz, 3H, -CH_2_CH_3_).


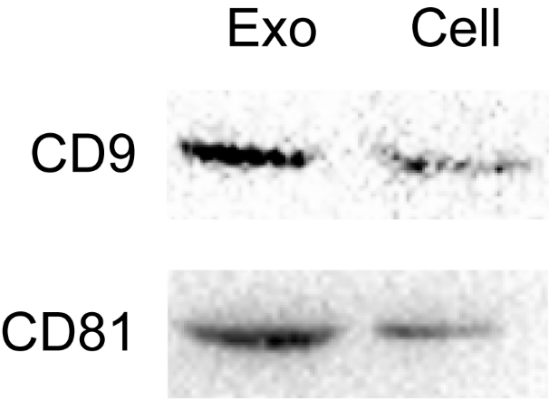


**Figure S2.** Detection of CD9 and CD81 proteins for 4T1-derived exosomes.


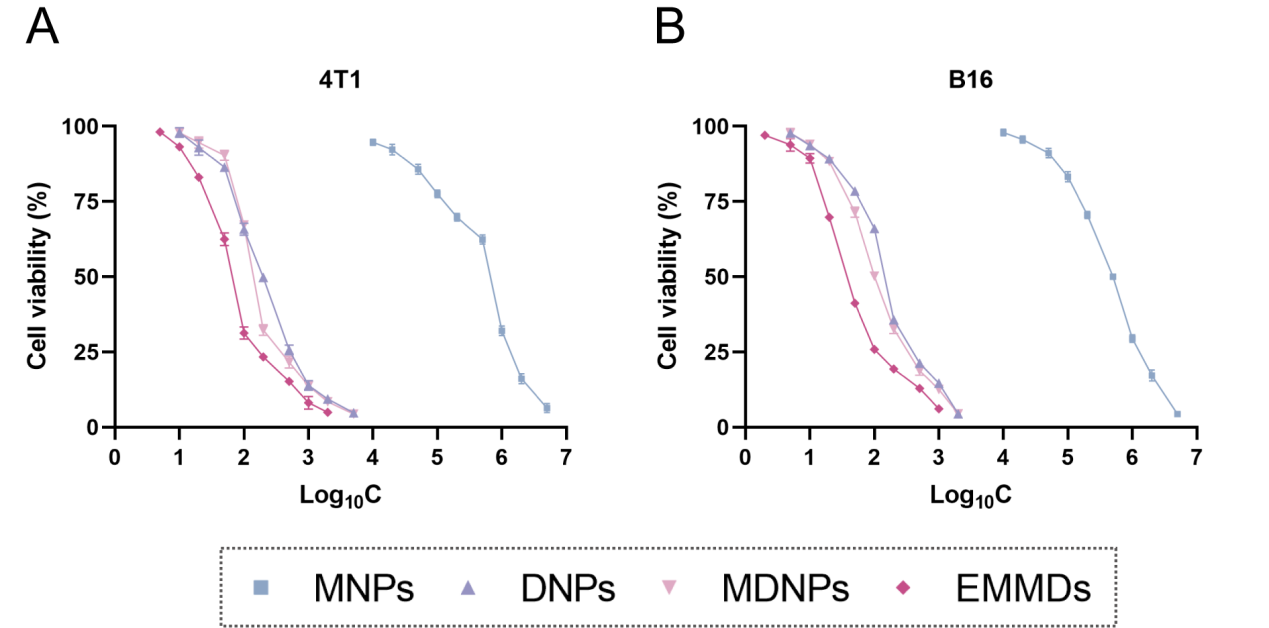


**Figure S3.** Cytotoxicity of MNPs, DNPs, MDNPs, and EMMDs against (A) 4T1 cells and (B) B16 cells for 48 h.


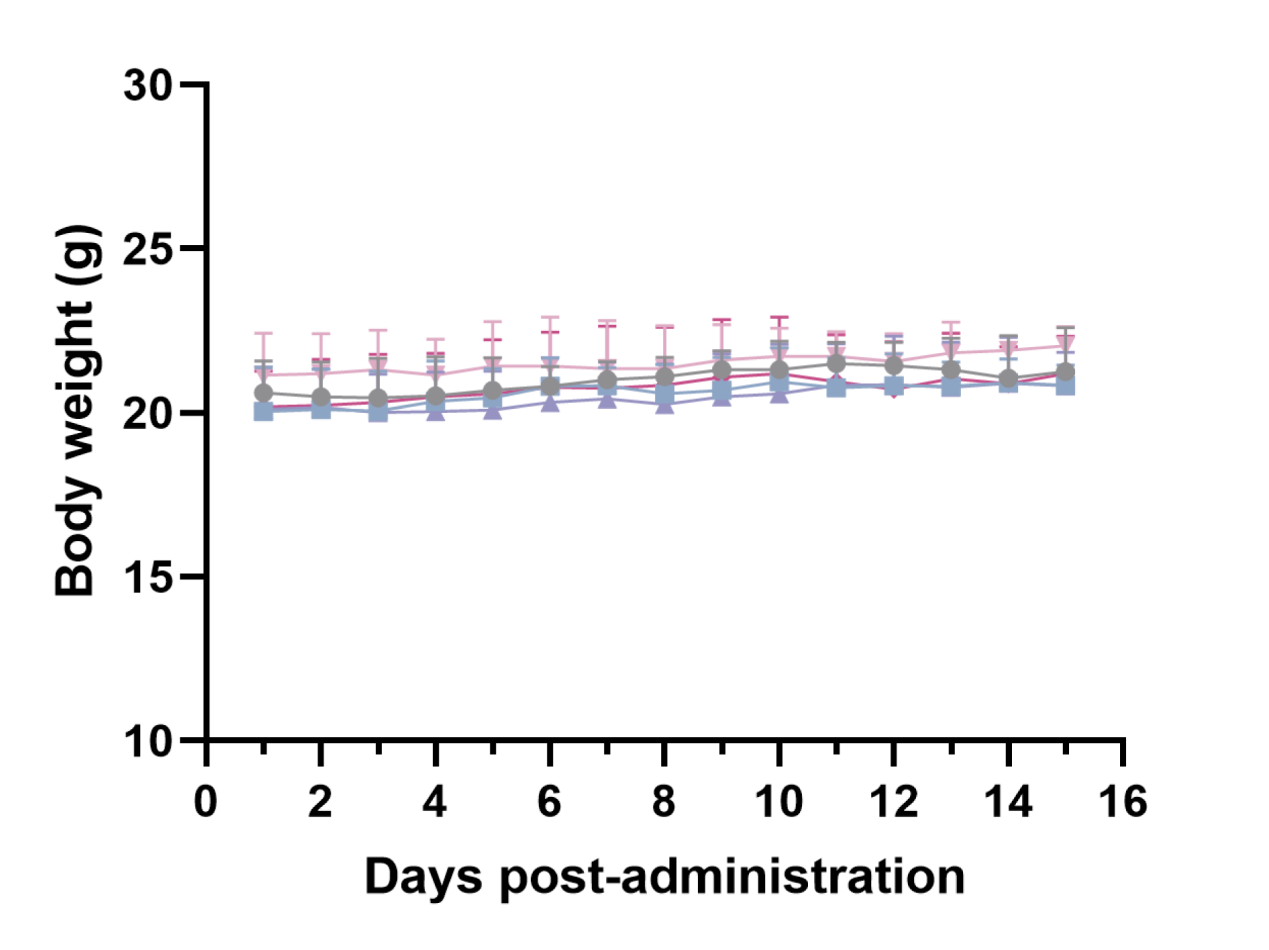


**Figure S4.** Changes in body weight of mice (n = 5).


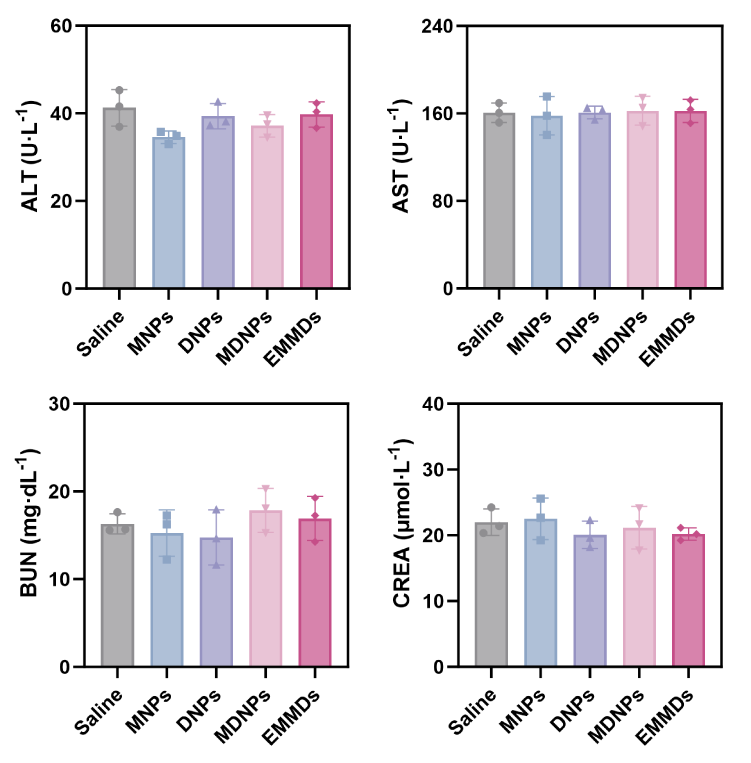


**Figure S5.** Hepatorenal function parameters. ALT: alanine aminotransferase (U·L^-1^), AST: aspartate aminotransferase (U·L^-1^), BUN: blood urea nitrogen (mg·dL^-1^), CREA: creatinine (μmol·L^-1^) (n = 3).


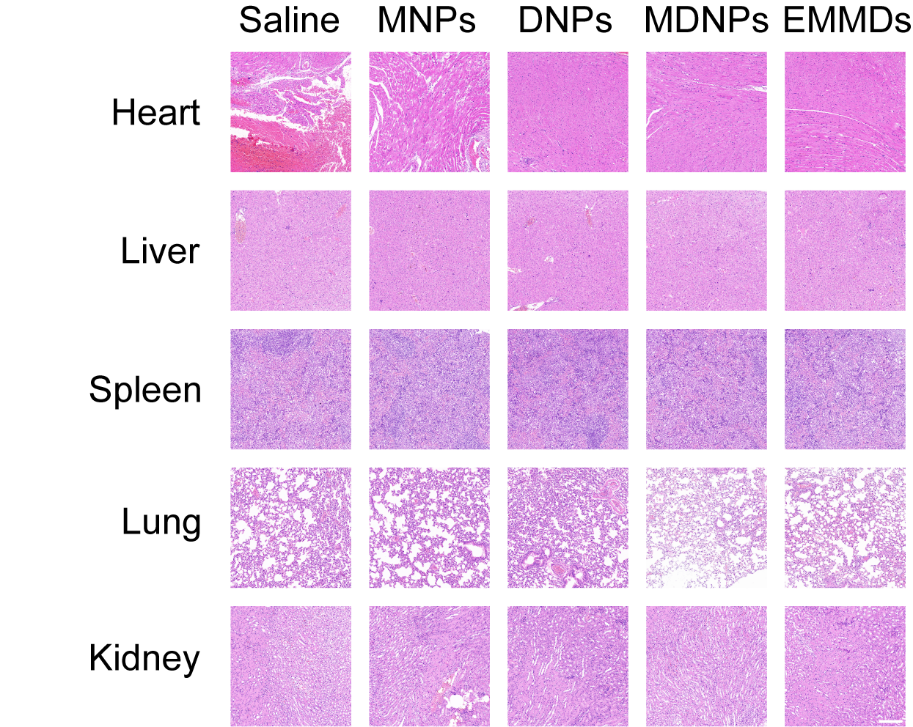


**Figure S6.** H&E staining of major organs of 4T1 tumor-bearing BALB/c mice after the last treatment. Scale bar = 100 μm.


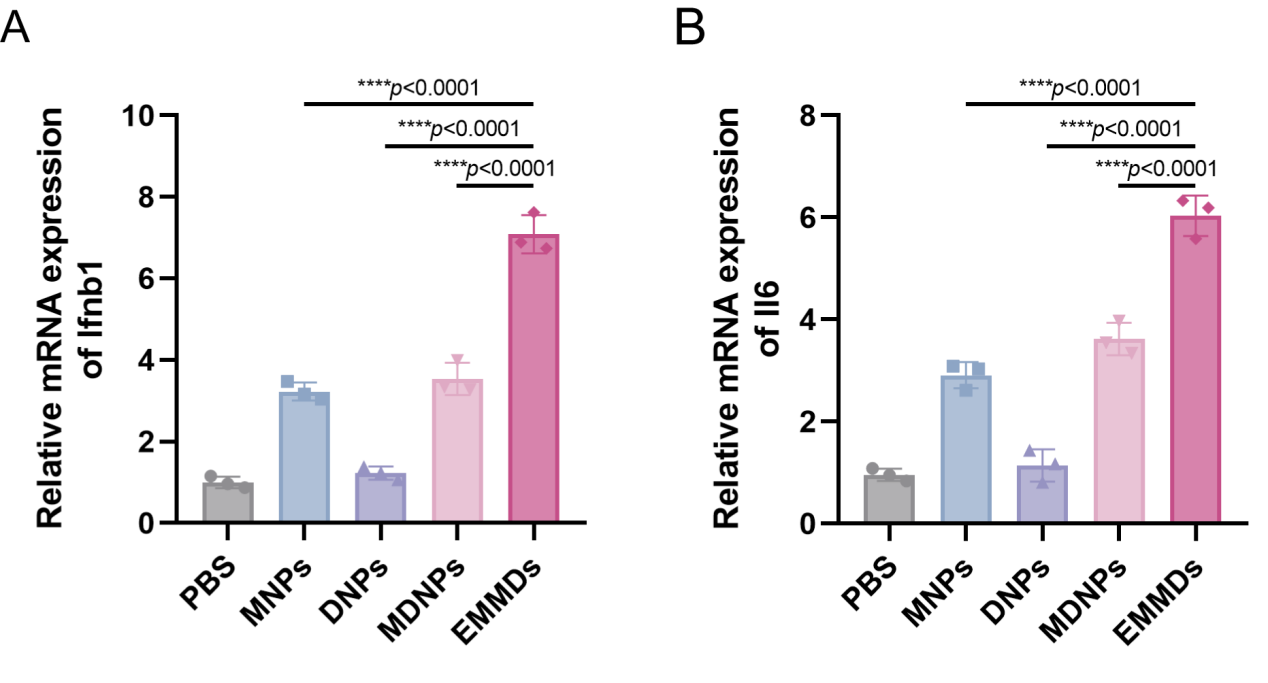


**Figure S7.** Quantification of abundance of mRNAs from tumor tissues for the indicated genes by RT-qPCR, n = 3. Statistical significance was determined using one-way analysis of variance (ANOVA), **P* < 0.05, ***P* < 0.01, ****p* < 0.001, *****p* < 0.0001, ns denotes no significant difference.
